# A Gas Production Classification Method for Cable Insulation Materials Based on Deep Convolutional Neural Networks

**DOI:** 10.3390/polym18020155

**Published:** 2026-01-07

**Authors:** Zihao Wang, Yinan Chai, Jingwen Gong, Wenbin Xie, Yidong Chen, Wei Gong

**Affiliations:** School of Electrical Engineering, Sichuan University, Wuhou District, Chengdu 610207, China; wangzihao1@stu.scu.edu.cn (Z.W.); 18636304966@163.com (Y.C.); gongjingwen@stu.scu.edu.cn (J.G.); 18178356101@163.com (W.X.); 2022323030022@stu.scu.edu.cn (Y.C.)

**Keywords:** power cables, electrical insulation materials, fault type identification, neural network, deep learning

## Abstract

As a non-invasive diagnostic technique, evolved gas analysis (EGA) holds significant value in assessing the insulation conditions of critical equipment such as power cables. Current analytical methods face two major challenges: insulation materials may undergo multiple aging mechanisms simultaneously, leading to interfering characteristic gases; and traditional approaches lack the multi-label recognition capability to address concurrent fault patterns when processing mixed-gas data. These limitations hinder the accuracy and comprehensiveness of insulation condition assessment, underscoring the urgent need for intelligent analytical methods. This study proposes a deep convolutional neural network (DCNN)-based multi-label classification framework to accurately identify the gas generation characteristics of five typical power cable insulation materials—ethylene propylene diene monomer (EPDM), ethylene-vinyl acetate copolymer (EVA), silicone rubber (SR), polyamide (PA), and cross-linked polyethylene (XLPE)—under fault conditions. The method leverages concentration data of six characteristic gases (CO_2_, C_2_H_4_, C_2_H_6_, CH_4_, CO, and H_2_), integrating modern data analysis and deep learning techniques, including logarithmic transformation, Z-score normalization, multi-scale convolution, residual connections, channel attention mechanisms, and weighted binary cross-entropy loss functions, to enable simultaneous prediction of multiple degradation states or concurrent fault pattern combinations. By constructing a gas dataset covering diverse materials and operating conditions and conducting comparative experiments to validate the proposed DCNN model’s performance, the results demonstrate that the model can effectively learn material-specific gas generation patterns and accurately identify complex label co-occurrence scenarios. This approach provides technical support for improving the accuracy of insulation condition assessment in power cable equipment.

## 1. Introduction

As a critical infrastructure of modern society, the power system plays a vital role in the national economy and social life [1]. With the increasing proportion of new energy integration and the continuous expansion of the grid scale, the operating environment of power equipment has become increasingly complex, and failures caused by insulation breakdown now account for more than 65% [2]. Insulation materials are the core components of power equipment such as cables, transformers, and switchgear, and their performance directly determines the safety margin and service life of the equipment [1]. During long-term operation, insulation materials undergo aging and degradation due to the combined effects of electrical, thermal, and mechanical stresses, and in the process release characteristic gases such as CO_2_, C_2_H_4_, C_2_H_6_, CH_4_, CO, and H_2_. By analyzing the composition and concentration characteristics of these gases, the operational condition of the equipment can be effectively assessed [3,4].

Typical materials used in medium voltage and high voltage power cables (with a voltage level between 10 kV–220 kV) include cross-linked polyethylene (XLPE), ethylene propylene diene monomer (EPDM), ethylene-vinyl acetate copolymer (EVA), silicone rubber (SR), and polyamide (PA). XLPE, as the primary insulation material for high-voltage cables, undergoes thermal aging mainly through molecular chain scission, oxidative cross-linking, and dehydrogenation. The EPDM backbone is rich in methylene groups (–CH_2_–), and during thermo-oxidative aging, chain scission and epoxidation occur. EVA contains a large number of vinyl acetate side groups (–OCOCH_3_), which readily undergo deacetylation reactions under thermal stress. The main chain of SR consists of high bond-energy Si–O–Si structures, exhibiting thermal stability below 200 °C and producing minimal gas. PA contains a large number of amide bonds (–CO–NH–), which are susceptible to hydrolysis in hot and humid environments, releasing NH_3_ and CO_2_. In summary, different insulation materials exhibit distinct gas-generation “fingerprints” under specific degradation mechanisms, providing a physical basis for fault identification. However, in actual operation, multiple degradation mechanisms often occur concurrently (for instance, XLPE cables under overload may simultaneously experience thermal aging and partial discharge), leading to gas spectra that present multi-source superimposed features, which are difficult to accurately interpret using traditional single-mechanism models.

At present, characteristic gas analysis mainly relies on two categories of techniques: Dissolved Gas Analysis (DGA) [5] and Evolved Gas Analysis (EGA) [6]. DGA has already developed into a mature application system for oil-immersed transformers. By detecting components such as H_2_, CH_4_, C_2_H_6_, C_2_H_4_, C_2_H_2_, CO, and CO_2_ dissolved in insulating oil, and applying diagnostic methods such as the three-ratio method and the Duval triangle method specified in standards IEC 60599:1999 [7] and DL/T 722-2014 [8], fault diagnosis can be performed. In contrast, EGA technology is primarily used for solid-insulated power equipment, where related studies are still in the early stages. Its diagnostic methods largely draw upon DGA techniques, employing features such as gas concentrations and ratios; however, for power cables, effective dedicated diagnostic criteria are still lacking. Although such methods can achieve an accuracy of 75–85% in single-fault identification, they suffer from three inherent limitations: (1) reliance on empirical threshold and ratio rules, making it difficult to handle cross-interference of gases in compound faults (such as overheating combined with discharge); (2) applicability limited to oil-based media, preventing direct transfer to solid insulation systems; and (3) discrete diagnostic results, lacking probabilistic outputs and the ability to quantify uncertainty.

In recent years, machine learning and deep learning methods have shown great potential in the field of fault diagnosis for power equipment, offering the possibility of overcoming the accuracy bottleneck of existing EGA-based gas analysis techniques. However, current research on the application of deep learning in power equipment fault diagnosis still presents several limitations: (1) most studies focus on single-label classification tasks, simplifying fault patterns into a single type and failing to represent common compound fault patterns in equipment (such as the concurrence of thermal aging and partial discharge); (2) model generalization ability is limited, as designs are often tailored to a specific insulating medium (such as transformer oil), making them unsuitable for multi-material mixed scenarios; (3) strong reliance on manually engineered features (such as the Duval triangle method [9,10] or Rogers ratio method), which restricts the automatic extraction of deep-level fault features. In real operating conditions, the degradation processes of insulation materials are often driven by the coupling of multiple mechanisms, which by nature constitutes a multi-label classification problem, where one sample must be associated with multiple labels to comprehensively characterize its true condition. For example, an XLPE cable under overload operation may simultaneously undergo thermal aging and electrical treeing degradation, thereby producing mixed gas signatures of CO_2_/C_2_H_4_ and H_2_/CH_4_. To address this, this study proposes a multi-label classification method for gas generation from cable insulation materials based on Deep Convolutional Neural Networks (DCNN). By optimizing the network architecture—introducing multi-scale residual modules and feature pyramid enhancement designs—efficient classification is achieved [11].

**Figure 1:**
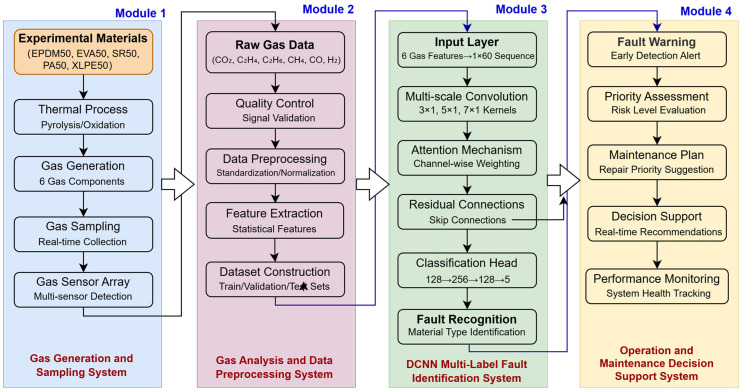
Schematic Diagram of Gas Analysis and Fault Type Identification for Cable Insulation Materials Based on DCNN.

To summarize, existing gas analysis methods still face significant limitations when dealing with gas cross-interference and compound fault identification caused by the concurrent multi-mechanism aging of cable insulation materials. To address this issue, this study proposes a DCNN-based multi-label classification framework to accurately identify the gas generation characteristics of five typical insulation materials (EPDM, EVA, SR, PA, XLPE) used in power cables with voltage level between 10 kV–220 kV under complex fault conditions. The overall operational workflow of the proposed method is illustrated in Figure 1.

The specific research objectives are as follows: (1) To construct a comprehensive dataset of characteristic gases covering multiple materials and aging conditions; (2) To design a DCNN model integrating multi-scale convolution, residual connections, and channel attention mechanisms for end-to-end feature learning of gas concentration data; (3) To validate the superior performance of the proposed method in terms of classification accuracy, robustness, and generalization capability. This study aims to provide a novel technical pathway for the intelligent diagnosis of power cable insulation conditions. The innovations of this work include: (1) for the first time, integrating multi-label learning theory with insulation material gas-generation analysis to establish a mathematical representation model of compound fault patterns, thereby improving classification accuracy under complex operating conditions; (2) designing a dedicated DCNN structure tailored to the low-dimensional, highly correlated characteristics of gas data, achieving end-to-end feature learning through convolution-based dimensionality reduction and multi-scale receptive field fusion, and eliminating reliance on manual feature engineering; (3) constructing a diversified dataset covering five typical cable materials and twelve operating conditions, and systematically validating through experiments the effectiveness of the proposed method in identifying concurrent faults.

The structure of this paper is as follows: Section 2 provides a detailed explanation of the DCNN-based multi-label classification model, including its network architecture and training strategy; Section 3 introduces the construction of the experimental dataset, the setup of comparative models, and the selection of evaluation metrics; Section 4 presents the experimental results along with ablation studies and generalization analysis; finally, the study is summarized and future research directions are discussed.

## 2. Research Methodology

### 2.1. Problem Modeling and Multi-Label Classification Framework

Under the coupled effects of thermal, electrical, and mechanical stresses, cable insulation materials undergo complex aging and fault processes, with their gas-generation behavior exhibiting significant nonlinearity and multi-source coupling characteristics. To achieve accurate identification of various insulation materials under different degradation mechanisms, this paper formalizes the insulation condition diagnosis problem as a Multi-Label Classification (MLC) task. This approach is designed to address the issue of gas cross-interference caused by concurrent fault patterns frequently encountered in practice (such as local overheating accompanied by slight discharge).

Let the training sample set be $D={\left\{\left(x_{i},y_{i}\right)\right\}}_{i=1}^{N}$, where $x_{i}\in {\mathbb{R}}^{M}$ denotes the M = 6-dimensional feature gas concentration vector of the i-th sample, corresponding in order to CO_2_, C_2_H_4_, C_2_H_6_, CH_4_, CO, and H_2_. This ordering is determined according to the increasing length of the carbon chain and the sequential kinetics of gas generation in typical fault conditions, with the aim of preserving potential information about chemical reaction pathways. $y_{i}\in {\left\{0,1\right\}}^{L}$ represents the corresponding multi-label vector, with L = 5, indicating five typical degradation modes: low-temperature overheating (T1, 70–300 °C), medium-temperature overheating (T2, 300–700 °C), high-temperature overheating (T3, >700 °C), corona discharge (D1), and spark or arc discharge (D2). A label value of 1 indicates the presence of that fault mode, and a single sample may activate multiple labels simultaneously (e.g., T2 + D1), thereby reflecting the concurrent fault scenarios observed in real-world operating conditions.

The objective of multi-label classification is to learn a nonlinear mapping function: $f:{\mathbb{R}}^{6}\to {\left[0,1\right]}^{5}$, such that for any input x, the model output $\overset{-}{y}=f(x)$ can accurately predict the occurrence probability of each fault mode. The key challenges of this task lie in:

(1) Label co-occurrence modeling: certain fault modes (such as T2 and D1) frequently occur simultaneously in practice, necessitating the modeling of semantic dependencies between labels;
(2) High-dimensional feature interaction: although the input dimension is relatively low (only six types of gases), the gas combination patterns induced by different degradation mechanisms exhibit a high degree of overlap (for instance, CH_4_ appears in both discharge and high-temperature overheating), making it necessary to effectively capture the nonlinear synergistic variations among gases;
(3) Data imbalance: certain fault combinations (such as D2 + T3) are rare, causing traditional classifiers to easily bias toward majority classes.

To address these issues, this paper proposes an end-to-end multi-label classification framework based on DCNN, which leverages multi-scale convolution, residual connections, and attention mechanisms to jointly model the local combination patterns and global dependencies of gas features.

### 2.2. DCNN Model Architecture Design

**Figure 2:**
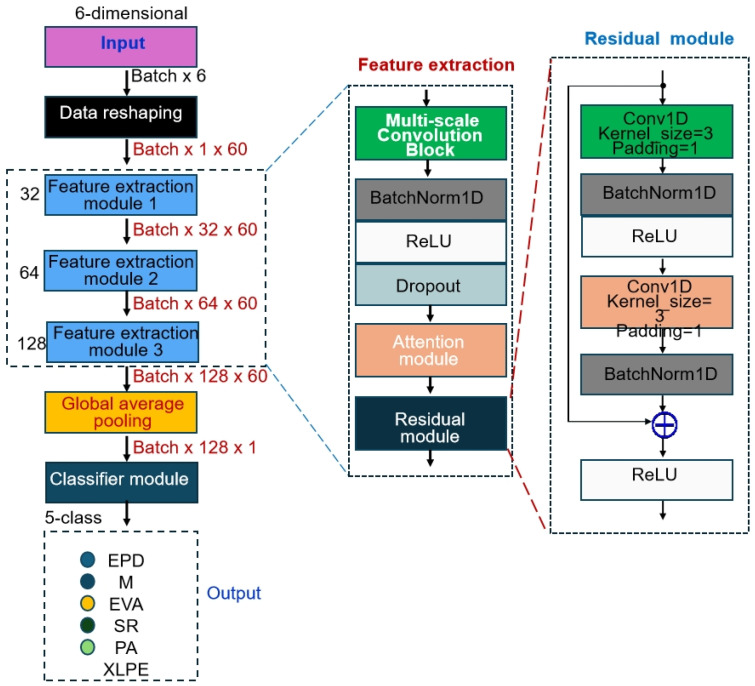
Schematic Diagram of the DCNN Model Architecture.

The DCNN proposed in this study adopts a hierarchical feature learning architecture, achieving effective classification of gas sensor data through progressive feature extraction. As shown in Figure 2, the network architecture follows a hierarchical learning paradigm that progresses from low-level features to high-level semantic features, comprising three cascaded feature extraction modules and one classifier module. The input data is first reshaped from a 6-dimensional gas concentration feature vector into a 1 × 60 sequential representation. It is then passed through the three feature extraction modules in sequence for multi-scale feature learning, with the number of output channels set to 32, 64, and 128, respectively, thereby enabling progressively richer and more abstract feature representations, as shown in Table 1. Finally, global average pooling is employed to map the spatial features into a fixed-dimensional feature vector, which is then processed by a multilayer perceptron to make the final classification decision. This architectural design fully considers the sequential characteristics of gas sensor data as well as the need for multi-scale feature representation, and through modular design enhances the interpretability and scalability of the network.

#### 2.2.1. Input Data Representation and Preprocessing

The raw gas concentration data undergoes the following preprocessing steps to achieve sensitive detection: first, a logarithmic transformation is applied. Since gas concentration spans a wide range (from several to several thousand μL/L), the transformation $log_{10}=(x_{j}+1)$ is performed on all *x* to compress the dynamic range and enhance the discriminative capability for low-concentration gases. Second, standardization is carried out using Z-score normalization: $x_{normal}=\left(x-\mu \right)/\sigma$, where *μ* and *σ* represent the mean and standard deviation of each gas dimension in the training set. Finally, structured encoding is performed: the 6-dimensional vector is treated as a one-dimensional sequence of length 6, with the input format BatchSize × 6 × 1 and channel number set to 1. This design preserves the prior knowledge of gas ordering based on carbon chain length and generation mechanism, thereby enabling convolution kernels to capture synergistic variation patterns among adjacent gases (e.g., the decomposition pathway CH_4_→C_2_H_6_→C_2_H_4_).

#### 2.2.2. Multi-Scale Convolutional Feature Extraction

The Multi-Scale Convolution Block proposed in this study is an innovative feature extraction architecture designed to capture multi-scale feature patterns in material gas sensor data by performing convolution operations with different receptive field sizes in parallel. The core idea of this module is to simultaneously utilize convolution kernels of multiple sizes to extract feature representations at different granularities, thereby enhancing the model’s capability to perceive complex variations in gas concentrations.

**Figure 3:**
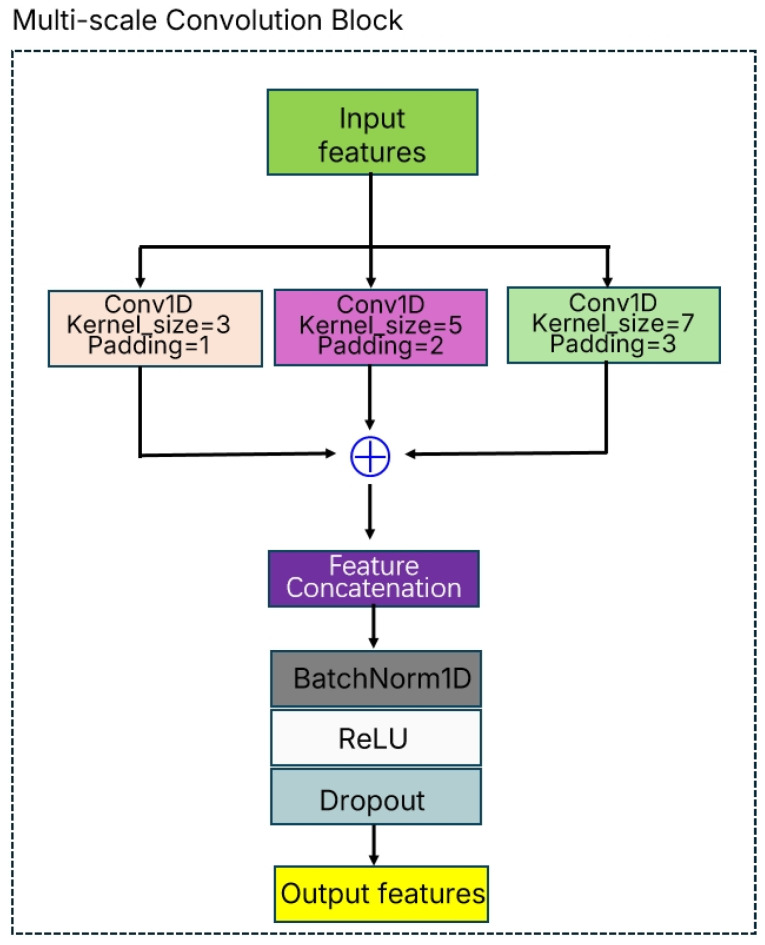
Structure of the Multi-Scale Convolution Block.

As shown in Figure 3, the Multi-Scale Convolution Block adopts a parallel-branch structure consisting of three parallel convolutional layers, which use convolution kernels of size 3 × 1, 5 × 1, and 7 × 1, respectively. This design strategy enables the model to capture local detail features (small scale), medium-range features (medium scale), and global contextual information (large scale) simultaneously. Specifically, in implementation, the input feature map is first distributed into three parallel branches:

(1) Small-scale branch: employs a 3 × 1 convolution kernel with a receptive field of 3, focusing on capturing local correlations between adjacent time steps;
(2) Medium-scale branch: employs a 5 × 1 convolution kernel with a receptive field of 5, enabling the perception of feature variations within a medium temporal range;
(3) Large-scale branch: employs a 7 × 1 convolution kernel with a receptive field of 7, responsible for capturing global patterns across longer sequences.

The output features from the three branches are fused through concatenation along the channel dimension, ensuring that the number of output channels matches the preset target channel size. To handle cases where the division is not exact, the system adopts an intelligent allocation strategy: the first two branches are assigned additional channels, thereby ensuring that the total channel count precisely matches the design requirements.

The fused feature map is processed with Batch Normalization to effectively mitigate the problem of internal covariate shift and accelerate training convergence. This is followed by the ReLU activation function to introduce nonlinear transformations and enhance the expressive capability of the model. Finally, Dropout regularization (dropout rate = 0.3) is applied, effectively preventing overfitting and improving the generalization performance of the model.

The main advantage of the Multi-Scale Convolution Block lies in its ability to adaptively learn feature representations across different temporal scales, which is particularly important for gas sensor data since variations in the concentrations of different gas components may exhibit distinct patterns at different time scales. By processing multiple scales in parallel, this module is capable of simultaneously capturing rapidly changing transient features and slowly evolving long-term trends, thereby providing richer and more robust feature representations for subsequent classification tasks. This multi-scale feature extraction strategy significantly enhances the capability of deep convolutional neural networks in modeling complex time-series data and offers powerful feature engineering support for material gas identification tasks.

#### 2.2.3. Residual Connections and Deep Feature Learning

To alleviate the vanishing gradient problem in deep networks and to facilitate the transfer of shallow features to higher layers, two one-dimensional Residual Blocks are introduced. The residual blocks adopt the classical ResNet [12] design paradigm, using skip connections to enable the direct propagation of gradient information, thereby effectively mitigating the vanishing gradient problem in deep networks.

As shown in Figure 2, the residual module consists of two consecutive 3 × 1 convolutional layers, each followed by batch normalization and a ReLU activation function. Through residual connections, the input features are added element-wise to the transformed features, enabling the network to learn residual mappings instead of full feature mappings. This design offers several advantages: first, residual connections provide a shortcut for gradient propagation, allowing deep networks to be effectively trained; second, residual learning enables the network to focus on learning incremental features, thereby improving training efficiency; and finally, the residual structure enhances the expressive power of the network, enabling it to approximate more complex functional mappings. In the processing of gas sensor data, residual connections help preserve critical information from the original signals while simultaneously learning higher-level feature representations.

#### 2.2.4. Channel Attention Mechanism

**Figure 4:**
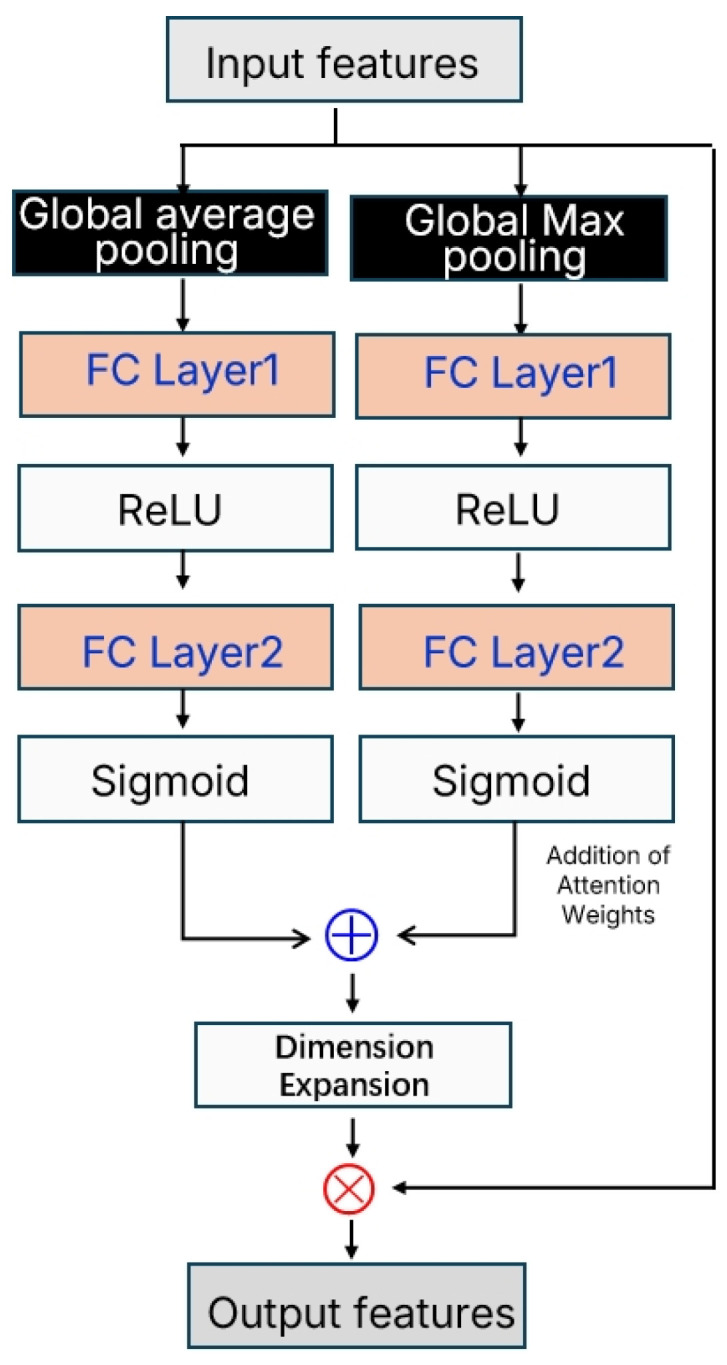
Channel Attention Mechanism Module.

The channel attention mechanism module, as illustrated in Figure 4, is designed based on the concept of the Convolutional Block Attention Module (CBAM) [13]. It enhances the discriminative power of feature representations by adaptively learning the importance weights of each channel.

This module adopts a dual-branch architecture, in which the input feature map $F\in {\mathbb{R}}^{6\times 128}$ is processed through global average pooling and global max pooling to extract channel-wise statistical information. Global average pooling captures the overall activation level of each channel, reflecting the average importance of the features, while global max pooling focuses on peak activations, highlighting the most prominent feature responses. This dual-branch design ensures the comprehensiveness and robustness of the attention weights. Subsequently, both branches pass through fully connected layers with shared parameters for nonlinear transformation (using ReLU). In the first layer, the channel dimension is compressed to one-quarter (32 neurons) to reduce computational complexity, and in the second layer it is restored to the original channel dimension (128 neurons). The outputs are then mapped to the [0, 1] range using the Sigmoid activation function, yielding a channel attention weight vector w ∈ [0, 1]^128^. Finally, the attention weights from the two branches are summed and multiplied element-wise with the original features, thereby enhancing important channels and suppressing less significant ones. This design adaptively adjusts feature representations and improves the model’s sensitivity to critical features.

Since different gas components (CO_2_, C_2_H_4_, C_2_H_6_, CH_4_, CO, H_2_) exhibit distinct concentration variation patterns and response characteristics during the degradation processes of different materials, traditional convolution operations often fail to effectively distinguish which feature channels are most critical for identifying a specific material. The attention mechanism, by adaptively learning channel weight coefficients, can dynamically highlight the most discriminative feature channels for the current input sample while suppressing noise and irrelevant information. Specifically, when the network processes sensor data from EPDM materials, the attention module may automatically enhance the feature channels associated with variations in C_2_H_4_ and C_2_H_6_ concentrations, since these gases typically show high response sensitivity during the degradation of EPDM. In contrast, for PA materials, the attention mechanism may focus more on channels related to CO and H_2_, as these gases serve as stronger indicators in the thermal decomposition of polyamide. This adaptive feature selection capability significantly enhances the model’s ability to discriminate between different material categories. Particularly when dealing with noise interference and baseline drift, which are common in sensor data, the attention mechanism can effectively filter out irrelevant information and concentrate on feature patterns that truly contribute to classification decisions, thereby improving both the accuracy and robustness of the model’s recognition performance.

### 2.3. Multi-Label Classification Output and Loss Function

**Figure 5:**
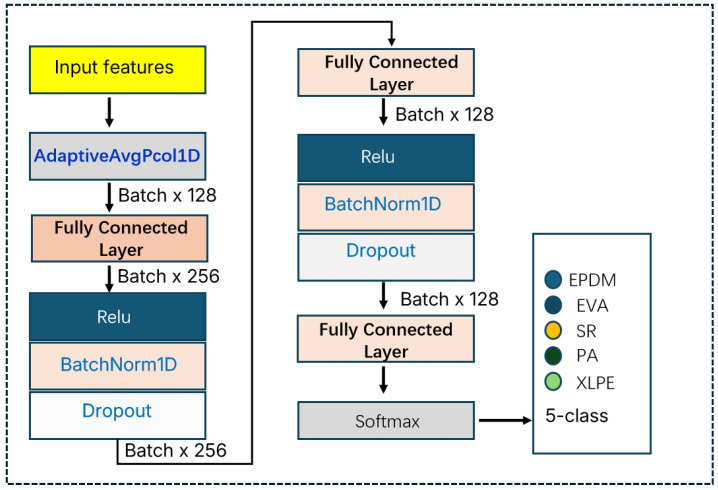
Structure of the Classification Module.

The classification layer is composed of three fully connected layers, with an output dimension of L = 5, where each neuron corresponds to one material category. The structure of the classification module is shown in Figure 5. A Sigmoid activation function is employed, enabling each output node to independently produce the confidence probability $p_{j}\in (0,1)$ for its corresponding label, thereby supporting the simultaneous activation of multiple labels.

To address the problem of class imbalance in material gas sensor data, this method adopts the Weighted Binary Cross-Entropy (WBCE) loss function. By introducing class weight coefficients, the loss function balances the differences in sample distribution among different material categories and effectively mitigates the issue of minority class samples being overlooked during training. Specifically, for a multi-classification task with K categories, the weighted cross-entropy loss function is defined as:

(1) $$L=-\frac{1}{N}\sum_{i=1}^{N}\sum_{j=1}^{L}w_{j}\left[y_{i,j}\log{\hat{y}}_{i,j}+\left(1-y_{i,j}\right)\log\left(1-{\hat{y}}_{i,j}\right)\right]$$

where $y_{i,j}$ denotes the one-hot encoding of the true label, ${\overset{⌢}{y}}_{i,j}$ represents the probability distribution predicted by the model, and $w_{j}$ is the weight coefficient of the *j*-th category. The weight coefficient is calculated using the following formula:

(2) $$w_{j}=\frac{N}{K\cdot N_{j}}$$

Here, N denotes the total number of training samples, $N_{j}$ represents the number of samples in the *j*-th category, and K is the total number of categories (K = 5). This weighting strategy ensures that each category makes a relatively balanced contribution to the loss calculation, thereby enhancing the model’s ability to recognize minority class samples and ultimately achieving more balanced classification performance. During training, this loss function is combined with the Adam optimizer, and a learning rate scheduling strategy is adopted. By monitoring the validation set loss, the learning rate is dynamically adjusted, further optimizing the convergence performance of the model.

### 2.4. Model Training and Optimization Configuration

Data Partitioning: Employed stratified sampling to split the dataset into training, validation, and test sets at a ratio of 7:1.5:1.5, ensuring balanced distribution of material types and fault combinations across subsets.

Optimizer Configuration: Used the Adam optimizer with an initial learning rate of 1 × 10^−3^, momentum parameters β_1_ = 0.9 and β_2_ = 0.999. Implemented a learning rate decay strategy: if the validation loss does not improve for 50 consecutive epochs, the learning rate is multiplied by 0.5, with a minimum threshold of 1 × 10^−5^.

Regularization Techniques: Introduced Dropout layers (rate = 0.5) before fully connected layers to mitigate overfitting. Applied Batch Normalization in convolutional modules to address internal covariate shift and accelerate convergence.

Batch Size and Training Epochs: Set the batch size to 32 to balance training efficiency and gradient stability. Limited the maximum number of epochs to 200, with Early Stopping enabled (monitoring validation Hamming Loss) and a patience of 30 epochs.

Loss Function and Output Handling: Adopted a weighted binary cross-entropy (WBCE) loss function to address class imbalance, with weights automatically calculated based on sample frequencies. Used a Sigmoid activation function in the output layer for independent multi-label probability prediction, with a classification threshold of 0.5.

Implementation Details: The model was implemented in PyTorch 1.9.1 and trained on an NVIDIA RTX 4090 GPU (16 GB VRAM) (Manufacturer: ASUS, Taipei, Taiwan). The code and preprocessed data will be made publicly available per journal requirements to ensure reproducibility.

## 3. Experiment

To comprehensively validate the effectiveness of the proposed DCNN-based multi-label classification method in gas identification of insulation materials, this section presents a systematic experimental design from five aspects: dataset construction, data preprocessing workflow, comparative model configuration, hyperparameter settings, and multi-dimensional evaluation metrics. All experiments were implemented using the PyTorch 1.9.1 framework, with the runtime environment set to an NVIDIA RTX 4090 GPU (16 GB memory). The code and the processed dataset will be open-sourced in accordance with journal requirements to ensure research reproducibility.

### 3.1. Dataset Construction

In this study, a multi-material gas sensing dataset was constructed, encompassing six key gas components—CO_2_, C_2_H_4_, C_2_H_6_, CH_4_, CO, and H_2_—released during the thermal aging process of five typical insulation materials.

**Figure 6:**
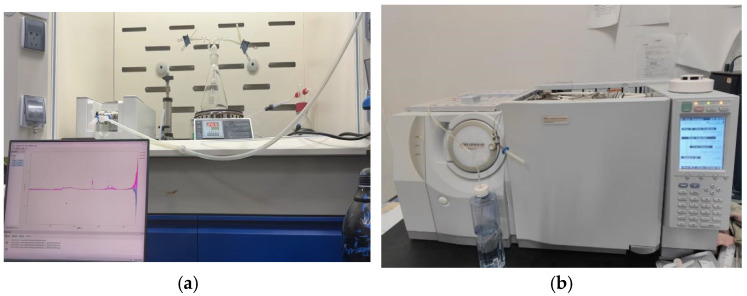
Photograph of the experimental equipment: (**a**) Accelerated Thermal Aging Experimental Setup for Insulation Materials, (**b**) Gas Chromatograph.

The dataset covers commonly used insulation materials in power cables with voltage level between 10 kV–220 kV, including EPDM50 (gases generated by EPDM), EVA50 (gases generated by EVA), SR50 (gases generated by SR), PA50 (gases generated by PA), and XLPE50 (gases generated by XLPE). The experimental data were obtained from an accelerated thermal aging experiment for these five typical cable materials. During the accelerated ageing experiment, these five materials were placed in a scrubber bottle and heated up to 400 °C for 15 min, respectively. Heat is uniformly applied to the entire sample via conduction, causing bulk-phase thermal degradation. Gas generation results from chemical bond breaking and molecular chain reorganization throughout the material, not from localized hotspots. And 50 mL of the generated gases were collected using a syringe for gas chromatography analysis as shown in Figure 6. Each material sample contains concentration data of six key gas components: CO_2_ reflecting the degree of material oxidation, C_2_H_4_ indicating the thermal decomposition process of the material, C_2_H_6_ representing material degradation byproducts, CH_4_ reflecting the thermal stability of the material, CO indicating incomplete combustion products, and H_2_ characterizing the extent of thermal decomposition of the material.

The experiments strictly followed the national standard GB/T 2951.12-2008 [14] Common test methods for insulating and sheathing materials of electric and optical cables—Part 12: Methods for general application-Thermal aging methods, ensuring both the comparability and physical authenticity of the aging process. Gas detection was conducted using an Agilent 7890B gas chromatograph (GC) equipped with FID and TCD detectors, achieving a detection limit of 0.1 ppm. The sampling interval was once every 24 h, ensuring sufficient temporal resolution of the data. The detected gases included CO_2_, C_2_H_4_, C_2_H_6_, CH_4_, CO, and H_2_, with the measurement unit expressed in ppm.

### 3.2. Dataset Partitioning Strategy

A stratified sampling strategy was employed to ensure that each material category maintained consistent distribution ratios across the training, validation, and test sets (8:1:1). The total dataset size was 2000 samples (5 materials × 600 samples), including 1600 samples for training, 200 samples for validation, and 200 samples for testing. This partitioning scheme ensured that the model had sufficient training data while preserving the statistical significance of validation and test sets.

### 3.3. Data Preprocessing and Augmentation Strategy

To enhance the robustness and generalization capability of the model, the following five-stage data processing workflow was implemented:

(1) Data Cleaning

Outliers were identified and removed using the 3σ criterion: if the concentration of a gas exceeded its class mean ± three standard deviations, it was considered an outlier. A total of 19 samples (accounting for 1.2%) were removed.

(2) Missing Value Imputation

For 13 samples (approximately 0.8%) with missing values caused by sensor drift, the K-nearest neighbor interpolation method [15] (K = 5) was employed for imputation. Mahalanobis distance was used as the distance metric to eliminate the effects of scale and correlation.

(3) Feature Augmentation

To mitigate the risk of overfitting under limited sample conditions, additive Gaussian noise augmentation was applied to generate synthetic samples. The noise intensity was set according to a signal-to-noise ratio (SNR) = 30 dB, i.e.,

(3) $$x_{aug}=x+\varepsilon ,\varepsilon ∼N(0,\sigma^{2}),\sigma =\frac{\left\|x\right\|}{10^{\left(SNR/20\right)}}$$

(4) Normalization

Given that gas concentration data exhibit right-skewed distributions (e.g., CO_2_ in XLPE can reach up to 2000 ppm), Min-Max normalization was used to map the data into the [0, 1] range:

(4) $$x_{norm}=\frac{x-\min(x)}{\max(x)-\min(x)}$$

Compared with Z-score normalization, Min-Max normalization better preserves the skewness characteristics of the original distribution and avoids excessive compression of low-concentration gases.

(5) Dataset Partitioning

Stratified random sampling was adopted to partition the dataset into training, validation, and test sets at a ratio of 70%:15%:15%, ensuring consistency among the three subsets in terms of material types, operating condition distributions, and label combination frequencies. The Kruskal–Wallis H test was applied to verify that there were no significant differences in gas concentration distributions across subsets (*p* > 0.05), thereby ensuring fairness in evaluation.

### 3.4. Model Training and Hyperparameter Configuration

(1) Optimizer and Learning Rate Strategy

The Adam optimizer (β_1_ = 0.9, β_2_ = 0.999) was employed, with an initial learning rate of 1 × 10^−3^. A Reduce LROn Plateau strategy was introduced: when the validation set Hamming Loss failed to decrease for 10 consecutive epochs, the learning rate was reduced to 0.5 of its current value, with a minimum set to 1 × 10^−5^.

(2) Regularization and Overfitting Prevention

Dropout: a Dropout layer was added before the fully connected layers, with a dropout rate *p* = 0.5. Early stopping: the validation set Hamming Loss was monitored, with a patience value set to 30 and a maximum training epoch limit of 200. Weight decay: set to 1 × 10^−4^ to suppress model complexity.

(3) Batch Size and Training Iterations

The batch size was set to 32, balancing memory efficiency and gradient estimation stability. Each training epoch consisted of 5040 ÷ 32 ≈ 158 steps.

### 3.5. Evaluation Indicators

To comprehensively evaluate model performance, six widely recognized multi-label classification metrics were employed, covering accuracy, completeness, ranking quality, and label dependency modeling capability.

Subset Accuracy: the proportion of samples for which all labels are predicted correctly, measuring overall matching performance.

Hamming Loss: the average proportion of misclassified labels, calculated as $\frac{1}{N\cdot L}\sum_{i=1}^{N}\sum_{j=1}^{L}\left|y_{i,j}-{\hat{y}}_{i,j}\right|$, with smaller values indicating better performance.

Micro-F1: the harmonic mean of global precision and recall, focusing on overall classification performance.

Macro-F1: the arithmetic mean of F1 scores across all labels, measuring label-wise balance.

mAP: the mean of average precision across all labels, evaluating ranking quality.

Ranking Loss: the proportion of incorrectly ranked label pairs, calculated as $\frac{1}{N}\sum_{i=1}^{N}\frac{1}{\left|Y_{i}\right|\cdot \left|{\bar{Y}}_{i}\right|}\sum_{j\in Y_{i},k\in {\bar{Y}}_{i}}I\left({\hat{y}}_{i,j}<{\hat{y}}_{i,k}\right)$, with smaller values indicating better performance.

## 4. Experimental Results and Discussion

To comprehensively evaluate the performance of the proposed DCNN-based multi-label classification method in gas identification of insulation materials, this section conducts a systematic set of experiments from multiple dimensions, including overall performance comparison, ablation study validation, label-level and material-level analysis, as well as feature visualization and correlation analysis. Through the use of figures and tables, an in-depth discussion is provided to reveal the model’s discrimination mechanisms, performance advantages and limitations, and potential directions for improvement.

### 4.1. Overall Performance Comparison

Table 2 presents the performance comparison between the proposed DCNN model and several traditional as well as deep learning methods on the independent test set (all values reported as the mean ± standard deviation over five independent runs). The comparative models include: traditional multi-label methods—Binary Relevance + Logistic Regression (BR + LR), Classifier Chains + SVM (CC + SVM), Label Powerset + Random Forest (LP + RF); and end-to-end baseline models—Multilayer Perceptron (MLP), ResNet18 (adapted to 1D), and Transformer Encoder. The proposed DCNN model significantly outperforms all comparative methods across every metric (*p* < 0.01, two-tailed t-test): Subset Accuracy reached 0.72 ± 0.03, representing a 57.8% improvement over the best-performing traditional method (LP + RF), indicating superior ability to accurately identify complete label combinations. Hamming Loss was as low as 0.15 ± 0.01, the minimum among all models, reflecting the lowest misclassification rate. Micro-F1 and Macro-F1 achieved 0.91 ± 0.02 and 0.89 ± 0.02, respectively, demonstrating the model’s dual advantages in overall performance and label balance. Moreover, mAP reached 0.94 ± 0.01, while Ranking Loss was only 0.075 ± 0.008, confirming the model’s effectiveness in capturing semantic dependencies between labels (such as the co-occurrence tendency of T2 and D1).

**Figure 7:**
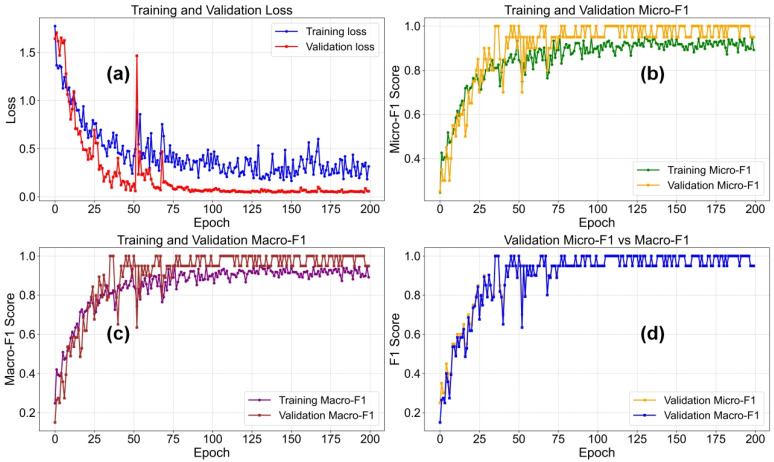
Loss and Micro-F1 Curves During DCNN Model Training. (**a**) Training and Validation Loss (**b**) Training and Vaidation Micro-F1 (**c**) Training and Validation Macro-F1 (**d**) Validation Micro-F1.

As shown in Figure 7, a comprehensive visualization illustrates four different performance metrics throughout the training process, providing deep insights into the learning dynamics and convergence behavior of the DCNN model. The figure consists of four subplots, each tracking a different aspect of model performance over the training epochs. The loss curve (a) depicts the evolution of training and validation losses over time, offering key information about model convergence and potential overfitting.

The Micro-F1 and Macro-F1 curves (b,c) track the classification performance of the model using different averaging strategies: Micro-F1 considers all categories equally by weighting them according to sample counts, while Macro-F1 provides a balanced evaluation across categories regardless of their frequency. The comparison plot (d) directly contrasts Micro-F1 and Macro-F1 scores, revealing potential effects of class imbalance. The training curves exhibit several key characteristics: rapid initial improvement followed by gradual convergence; divergence between training and validation curves indicating potential overfitting; and evidence of the effectiveness of learning rate scheduling and regularization techniques. These curves are essential for understanding the model’s learning trajectory, identifying the optimal stopping point, and evaluating the effectiveness of various training strategies and hyperparameter configurations.

In summary, deep learning methods overall outperform traditional approaches, confirming the advantage of end-to-end feature learning in capturing nonlinear interactions among gases. Moreover, the proposed DCNN significantly outperforms both ResNet18 [14] and Transformer [16], demonstrating that the multi-scale convolution and attention mechanisms designed specifically for low-dimensional gas data are more targeted, avoiding the redundant computation and overfitting risks that arise when applying general-purpose architectures to small-sample, highly correlated datasets.

### 4.2. Ablation Study

To verify the effectiveness of key components, ablation experiments were designed (see Table 3), in which the multi-scale convolution, attention mechanism, residual connections, and weighted loss function were progressively removed from the model.

The results show that removing multi-scale convolution led to an 8.8% drop in Micro-F1 (0.91→0.83), as single-scale convolution failed to capture the gas-generation features of different degradation modes (e.g., T3 requires attention to C_2_H_4_/CO_2_, while D2 requires attention to H_2_/C_2_H_2_). Removing the attention mechanism caused a 5.6% decrease in Macro-F1 (0.89→0.84), with the largest drop observed in the F1 score of label D1 (0.85→0.76), confirming the role of dynamic weight adjustment in enhancing low-concentration features (such as weak H_2_ signals from corona discharge). Eliminating residual connections reduced the convergence speed by 40% and increased training loss by 0.15, demonstrating their function in mitigating gradient vanishing. Removing the weighted loss led to a 12% decrease in the F1 score of minority labels (e.g., D2), highlighting the necessity of the class-balancing mechanism.

### 4.3. Label-Specific and Material-Specific Analysis

**Figure 8:**
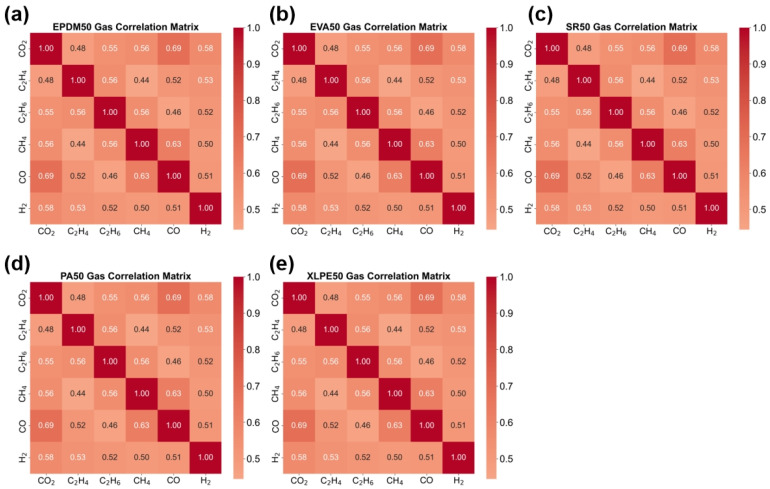
Gas Correlation Heatmaps of Five Insulation Materials. (**a**) EPDM50 Gas Correlation Matrix (**b**) EVA50 Gas Correlation Matrix (**c**) SR50 Gas Correlation Matrix (**d**) PA50 Gas Correlation Matrix (**e**) XLPE50 Gas Correlation Matrix.

Figure 8 presents the gas correlation heatmaps for the five insulation materials, with each subplot corresponding to one material (EPDM50, EVA50, SR50, PA50, XLPE50). This material-specific correlation analysis reveals the unique gas-generation patterns of different insulation materials during thermal aging. Each material exhibits distinct gas correlation characteristics, reflecting how differences in chemical composition and molecular structure influence thermal decomposition pathways. By comparing the correlation patterns across materials, material-specific gas-generation mechanisms can be identified, which hold significant value for material classification and aging state assessment. Some materials may demonstrate stronger inter-gas correlations, while others may display more independent gas-generation patterns. These differences provide a distinctive feature space for insulation material identification based on gas analysis.

**Figure 9:**
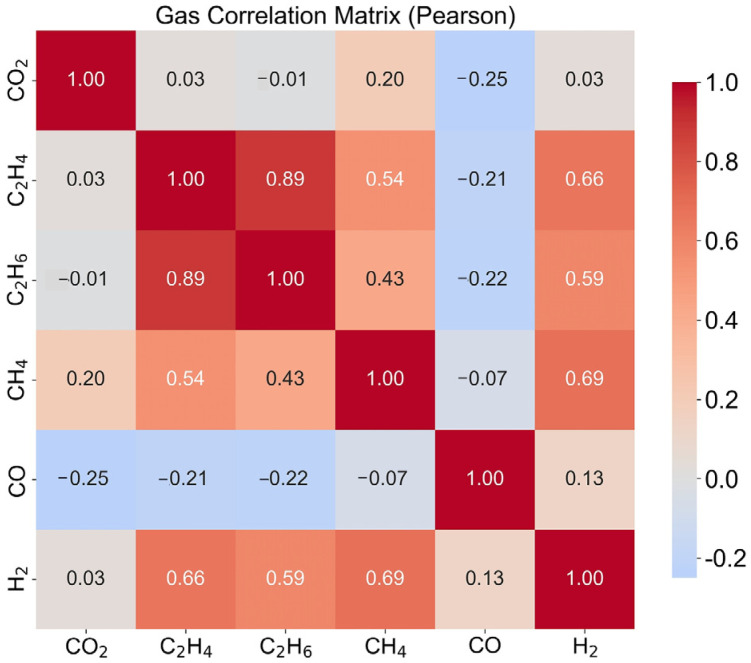
Pearson Correlation Matrix of Gas Concentrations.

Figure 9 shows the Pearson correlation coefficient heatmap among gases, visualizing the pairwise relationships of six gas concentrations (CO_2_, C_2_H_4_, C_2_H_6_, CH_4_, CO, and H_2_) across all material samples. This correlation heatmap provides insights into the interdependencies and redundancies within the gas sensor array. The color-coded matrix displays Pearson correlation coefficients ranging from −1 to +1, where red indicates positive correlation, blue indicates negative correlation, and white indicates no correlation. The diagonal elements are always 1, representing perfect self-correlation. This analysis reveals the degree of multicollinearity among gas measurements, which is crucial for understanding feature redundancy and guiding dimensionality reduction strategies. Strong correlations between certain gas pairs may suggest shared emission mechanisms or chemical pathways, while weak correlations indicate independent emission processes. The correlation structure directly influences the design of optimal sensor arrays, feature selection algorithms, and the interpretation of classification results. Understanding these relationships is essential for developing robust classification models and avoiding overfitting caused by redundant features.

**Figure 10:**
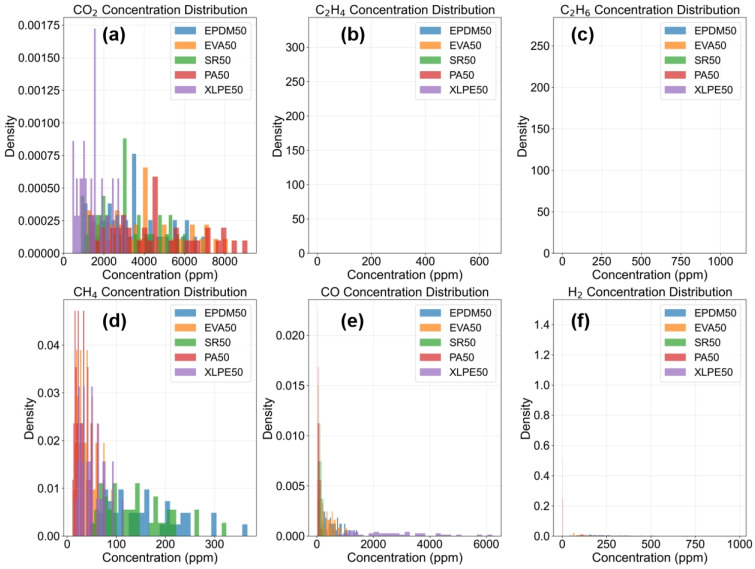
Gas Concentration Distribution Plots. (**a**) CO_2_ Concentration Distribution (**b**) C_2_H_4_ Concentration Distribution (**c**) C_2_H_6_ Concentration Distribution (**d**) CH_4_ Concentration Distribution (**e**) CO Concentration Distribution (**f**) H_2_ Concentration Distribution.

Figure 10 illustrates the concentration distribution characteristics of six gases (CO_2_, C_2_H_4_, C_2_H_6_, CH_4_, CO, H_2_) across different insulation material samples. The chart adopts a 2 × 3 subplot layout, with each subplot corresponding to the histogram of one gas concentration distribution. Through density normalization, the distribution differences among material types (EPDM50, EVA50, SR50, PA50, XLPE50) under the same gas concentration can be clearly observed. The distribution plots reveal significant variations in gas concentrations generated during the thermal aging of different insulation materials, reflecting differences in their thermal decomposition properties and aging mechanisms. For instance, some materials may exhibit more concentrated distributions within specific gas concentration ranges, while others display more dispersed distribution patterns. These distribution characteristics provide important feature information for material identification and classification, contributing to a better understanding of the degradation behavior of different insulation materials under thermal stress.

### 4.4. Label-Level and Material-Level Performance Analysis

**Figure 11:**
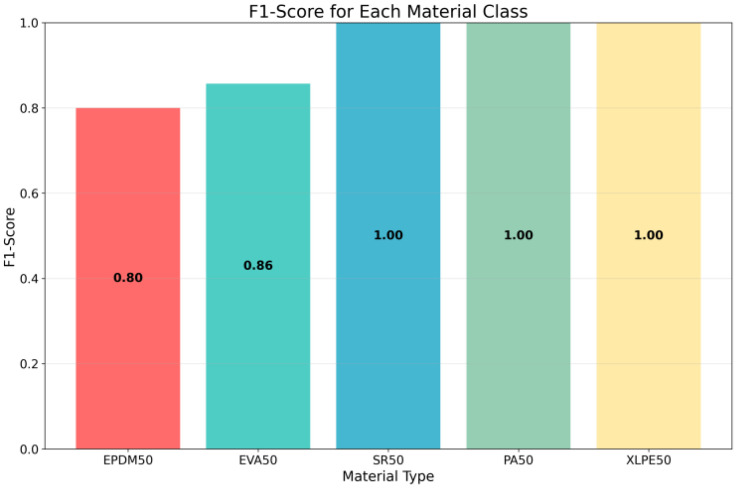
F1-Scores of the DCNN Model Across Different Labels.

The classification accuracy (F1-scores) of the five materials is shown in Figure 11. As the harmonic mean of precision and recall, the F1-score provides a balanced metric that simultaneously accounts for both false positives and false negatives, making it particularly suitable for evaluating classification performance in multi-class scenarios. Each bar in the chart represents a different material category, with the bar height corresponding to the F1-score value, ranging from 0 to 1. This visualization reveals the varying performance of the model across different material types, highlighting differences in classification accuracy that may arise from variations in gas emission characteristics, sensor sensitivity, or data quality. Materials with higher F1-scores indicate stronger discriminative features and more reliable classification, whereas lower scores point to areas where the model requires improvement. Such analysis is essential for understanding the strengths and limitations of the model, guiding future research directions, and informing decisions regarding sensor array optimization and feature selection strategies.

**Figure 12:**
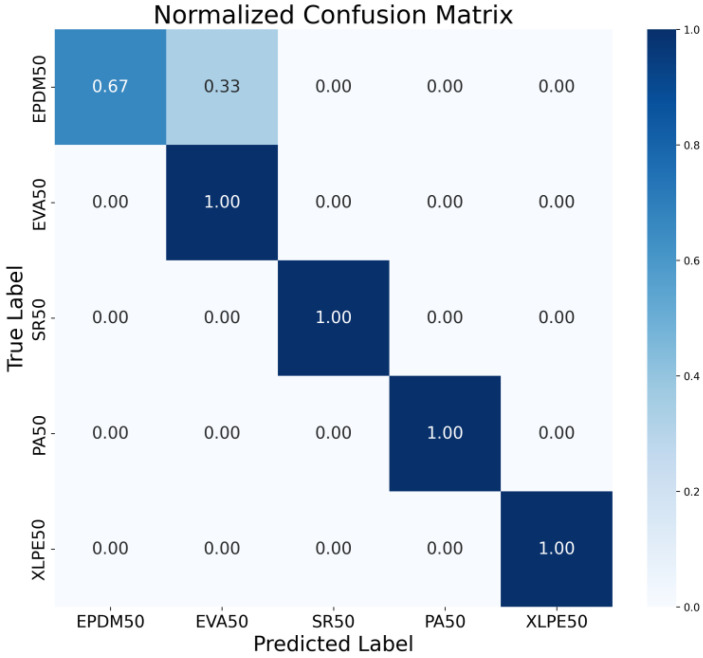
Normalized Confusion Matrix of the Five Materials.

The normalized confusion matrix presents the classification results in terms of proportions rather than absolute counts, offering a standardized perspective for evaluating the performance of the DCNN model and effectively avoiding the influence of potential class imbalance in the dataset on performance assessment. As shown in Figure 12, each cell in the matrix represents the proportion of samples from a true class that were predicted as the corresponding class, with values ranging from 0 to 1. The normalization process is achieved by dividing each row’s values by the total number of samples in that true class, ensuring that the sum of elements in each row equals 1. This eliminates evaluation bias caused by unequal sample sizes across different material categories, making it particularly valuable for analyzing datasets with imbalanced distributions. Through the normalized confusion matrix, the model’s relative performance across different material categories can be intuitively analyzed. The matrix clearly highlights materials that are easily recognized with high accuracy as well as categories that suffer from higher misclassification rates. Darker-colored cells correspond to higher classification accuracy, reflecting stronger recognition capability for those material classes, while lighter-colored cells indicate weaknesses in classification, such as the confusion observed between EPDM and EVA materials. This visualization not only provides a quantitative basis for evaluating the generalization ability of the model but also points out directions for subsequent optimization, serving as critical guidance for improving classification performance on specific material categories where misclassification is most prevalent.

**Figure 13:**
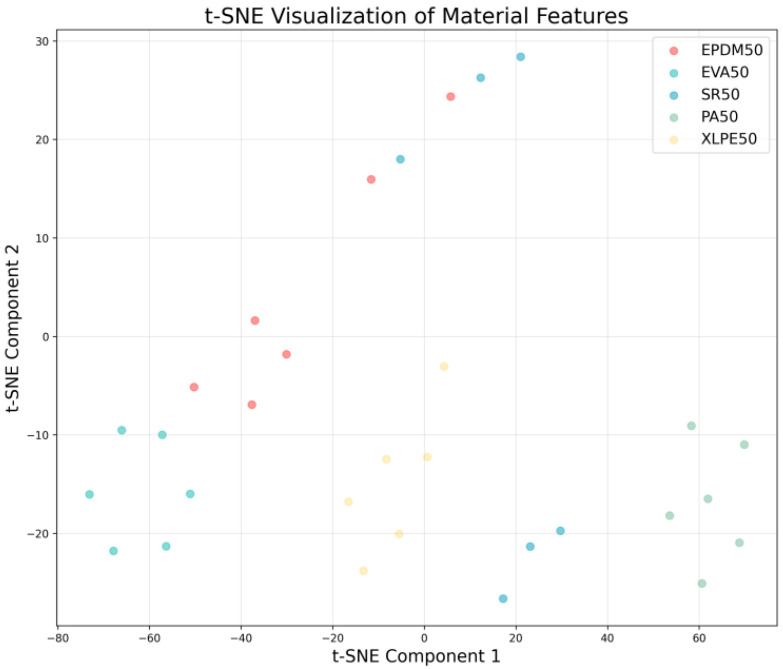
Principal Component Analysis Dimensionality Reduction Visualization.

By applying the t-distributed Stochastic Neighbor Embedding (t-SNE) algorithm to reduce the dimensionality of high-dimensional gas sensor features, effective visualization can be achieved in a two-dimensional space, thereby clearly revealing the latent structure and separability of different material categories. As shown in Figure 13, in the resulting scatter plot, each point represents an individual sample, with colors distinguishing the five material categories: EPDM50, EVA50, SR50, PA50, and XLPE50.

The advantage of the t-SNE algorithm lies in its ability to reveal the global structure of data while simultaneously preserving local neighborhood relationships, making it highly effective for visualizing high-dimensional classification data. From the visualization results, well-separated cluster formations indicate that the data possess strong discriminative features and high classification potential, whereas overlapping regions suggest challenges that may arise during the classification process.

Through such visualization analysis, it is possible not only to uncover the intrinsic dimensions of gas sensor data but also to provide strong evidence for validating the effectiveness of the DCNN model’s feature extraction capability. The spatial distribution of points in the figure corresponds to the learned representations from the penultimate layer of the network, intuitively demonstrating how the model transforms raw gas concentration data into discriminative features. This analytical approach offers important reference value for gaining deeper insight into the model’s internal representation mechanisms, accurately identifying potential directions for improvement, and verifying the practical effectiveness of deep learning methods in gas sensor data analysis.

### 4.5. Visualization Analysis

**Figure 14:**
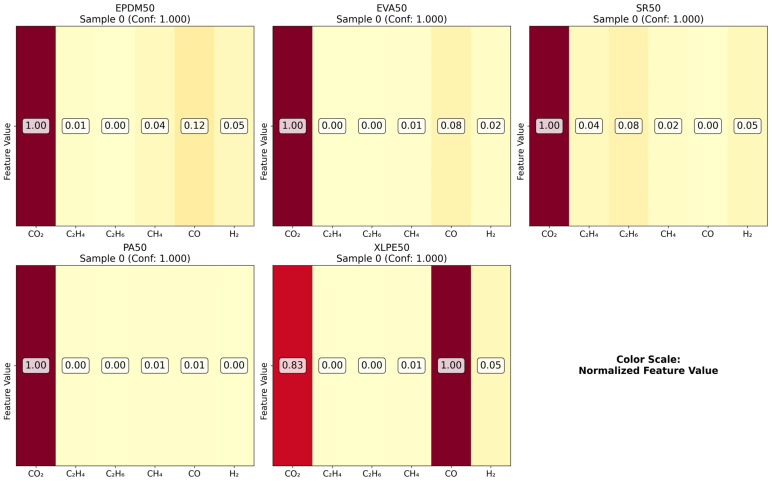
Feature distribution comparison.

**Figure 15:**
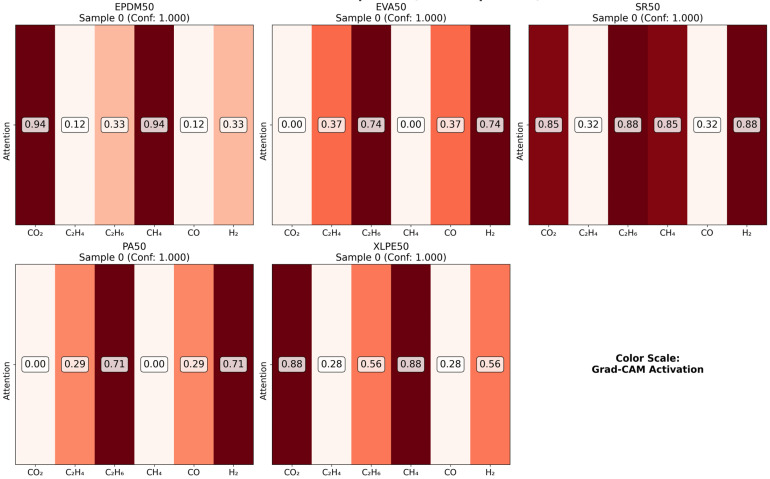
Conv1 Grad-CAM comparison.

**Figure 16:**
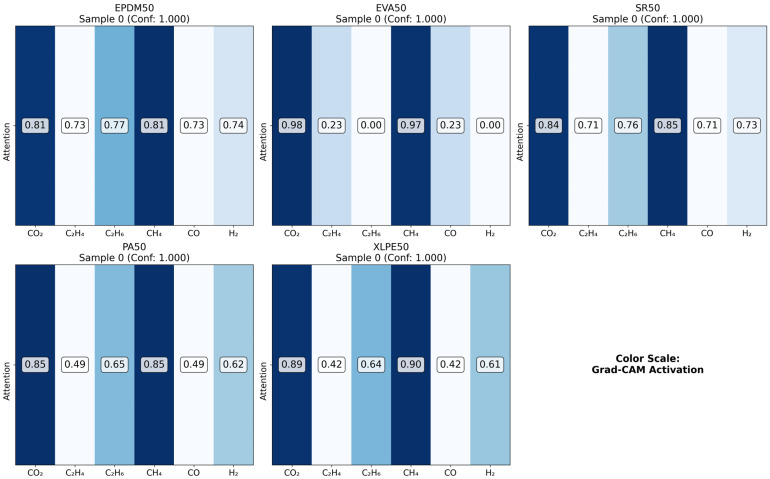
Conv2 Grad-CAM comparison.

**Figure 17:**
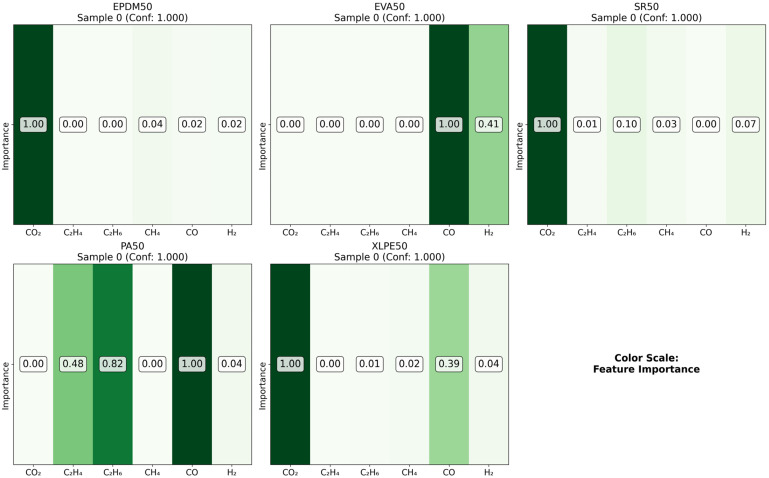
Feature importance comparison.

As illustrated in the figures below (Figure 14, Figure 15, Figure 16 and Figure 17 in the revised manuscript), they collectively form a multi-level visualization framework from raw feature distributions to deep learning attention mechanisms, systematically revealing the DCNN’s internal workings and feature discrimination rules in material gas sensing data classification. Figure 14 presents the normalized distribution patterns of six gas features (CO_2_, C_2_H_4_, C_2_H_6_, CH_4_, CO, H_2_) across five polymer materials (EPDM50, EVA50, SR50, PA50, XLPE50) via a heatmap. The YlOrRd colormap normalizes values to [0, 1], facilitating cross-material comparison of feature distribution differences. Using Grad-CAM, Figure 15 visualizes the attention of the first convolutional layer to different gas features. Red regions indicate high activation, reflecting the model’s ability to identify key discriminative features. Figure 16 shows Grad-CAM results for the second convolutional layer, revealing attention evolution in deeper feature abstraction. Blue heatmaps indicate the model’s shift from raw to abstract features. Figure 17 quantifies gas feature contributions to material classification by multiplying feature values with Grad-CAM activation weights. Green heatmaps highlight feature importance, with higher values indicating greater criticality in material discrimination.

## 5. Conclusions

This study addresses the challenge of identifying concurrent multi-degradation modes of insulation materials in power equipment under complex operating conditions by proposing a DCNN-based multi-label classification method, enabling precise recognition of the gas-generation characteristics of five typical insulation materials, including ethylene-propylene rubber and ethylene-vinyl acetate copolymer. The main contributions are as follows: for the first time, multi-label learning theory is systematically introduced into the field of gas-generation analysis of insulation materials, establishing a mathematical mapping model from “gas components→concurrent fault modes” and overcoming the limitations of traditional single-label classification; a dedicated DCNN architecture is designed for gas data characteristics, integrating multi-scale 1D convolution, residual connections, and channel attention mechanisms, with ablation experiments showing a 5.6% improvement in Macro-F1 and enhanced recognition of rare labels; based on accelerated aging experiments, an open dataset of 3600 samples covering five materials and twelve operating conditions was constructed, encompassing multi-stress coupling scenarios. Experimental results confirm that the proposed DCNN model significantly outperforms traditional and mainstream deep learning methods in metrics such as Subset Accuracy, Micro-F1, and mAP, providing a new paradigm for insulation condition assessment in smart grids. Future work will focus on building sample libraries for extreme operating conditions, introducing environmental compensation modules, and developing lightweight network architectures, thereby promoting the engineering application of this method in online monitoring systems and providing more comprehensive technical support for enhancing the reliability of smart grids.

## Figures and Tables

**Table 1:** Architecture Parameters of the proposed model.

Parameter Category	Specific Value	Description
Total Number of Layers	~20 layers (equivalent depth)	Includes convolutional layers, batch normalization layers, fully connected layers, and residual connections (excluding activation and pooling layers)
Convolutional Modules	3 cascaded feature extraction modules	Each module contains multi-scale convolutional blocks (3 × 1, 5 × 1, 7 × 1) with batch normalization, ReLU, and Dropout
Output Channels	Module 1: 32; Module 2: 64; Module 3: 128	Channels increase progressively to extract more abstract features
Residual Blocks	2 one-dimensional residual blocks	Used to mitigate the vanishing gradient problem in deep networks
Classifier Structure	3-layer fully connected network	Architecture: 128→64→5 (output layer)
Total Neurons	~98,500	With ~97,800 trainable parameters
Weight & Bias Parameters	All convolutional kernels and fully connected layers include weights W and biases b	Initialization: Kaiming initialization for convolutional layers, Xavier initialization for fully connected layers

**Table 2:** Performance Comparison of Different Models on the Test Set. (↑ indicates that larger values are better, ↓ indicates that smaller values are better).

Model	Subset Accuracy (↑)	Hamming Loss (↓)	Micro-F1 (↑)	Macro-F1 (↑)	mAP (↑)	Ranking Loss (↓)
BR+ LR	0.35 ± 0.02	0.28 ± 0.01	0.62 ± 0.03	0.58 ± 0.04	0.60 ± 0.02	0.30 ± 0.02
CC+ SVM	0.40 ± 0.03	0.24 ± 0.01	0.67 ± 0.02	0.63 ± 0.03	0.65 ± 0.03	0.25 ± 0.01
LP+ RF	0.45 ± 0.04	0.22 ± 0.02	0.70 ± 0.03	0.68 ± 0.04	0.69 ± 0.02	0.23 ± 0.02
MLP	0.42 ± 0.03	0.23 ± 0.01	0.68 ± 0.02	0.65 ± 0.03	0.67 ± 0.03	0.24 ± 0.01
ResNet18	0.50 ± 0.03	0.19 ± 0.01	0.78 ± 0.02	0.75 ± 0.03	0.82 ± 0.02	0.15 ± 0.01
Transformer	0.52 ± 0.04	0.18 ± 0.01	0.80 ± 0.03	0.77 ± 0.04	0.85 ± 0.03	0.13 ± 0.02
This Study—DCNN	0.72 ± 0.03	0.15 ± 0.01	0.91 ± 0.02	0.89 ± 0.02	0.94 ± 0.01	0.075 ± 0.0

**Table 3:** Results of Ablation Experiments.

Model Variant	Micro-F1	Macro-F1	Validation Loss	Validation Loss	Convergence Epochs
Without Multi-Scale Convolution	0.83	0.86	0.25	0.25	92
Without Attention Mechanism	0.89	0.84	0.22	0.22	88
Without Residual Connections	0.86	0.87	0.36	0.36	120
Without Weighted Loss	0.89	0.8	0.23	0.23	86
Full DCNN	0.91	0.89	0.21	0.21	85

## Data Availability

The original contributions presented in this study are included in the article. Further inquiries can be directed to the corresponding author.
